# Serious Bacterial Infection Risk in Febrile Infants Aged ≤90 Days With COVID-19

**DOI:** 10.7759/cureus.87938

**Published:** 2025-07-14

**Authors:** Rufu Furuya, Shun Kishibe, Takahiro Itagaki, Rei Miyake, Norihiro Tokuma, Yuta Matsumoto, Meiwa Shibata, Hiroshi Hataya

**Affiliations:** 1 Department of General Pediatrics, Tokyo Metropolitan Children's Medical Center, Tokyo, JPN; 2 Department of Emergency Medicine, Tokyo Metropolitan Children's Medical Center, Tokyo, JPN; 3 Department of Neonatology, Tokyo Metropolitan Children's Medical Center, Tokyo, JPN; 4 Department of Pediatrics, Tokyo Metropolitan Toshima Hospital, Tokyo, JPN; 5 Department of Hematology and Oncology, Tokyo Metropolitan Children's Medical Center, Tokyo, JPN; 6 Department of Infectious Diseases, Tokyo Metropolitan Children's Medical Center, Tokyo, JPN

**Keywords:** covid-19, febrile infant, sars-cov-2, sbi, serious bacterial infection, urinary tract infection, uti

## Abstract

Background

This retrospective cohort study compared the risk of serious bacterial infection (SBI) in febrile infants aged ≤90 days with and without COVID-19. While some studies have suggested that COVID-19-positive febrile infants may have a lower risk of SBI, this remains unclear, particularly in Japan. We investigated the incidence of SBI and clinical characteristics in COVID-19-positive versus COVID-19-negative febrile infants.

Methods

Febrile infants aged ≤90 days who visited the emergency department of the children's hospital in Japan between April 2021 and November 2022 were enrolled. The subjects were divided into a COVID-19 group, defined as subjects with positive polymerase chain reaction (PCR) findings for SARS-CoV-2 or those with close contact with a SARS-CoV-2 PCR-positive family member. SBI was defined as urinary tract infection (UTI), bacterial pneumonia, bacteremia, or bacterial meningitis.

Results

In total, 396 patients were included; of these, 125 patients were in the COVID-19 group. SBI was diagnosed in 7/125 patients (5.6%) of this group vs. 45/271 patients (16.6%) of the non-COVID-19 group (p<0.05). All cases of SBI diagnosed in the former were of UTI. No notable differences in respiratory or gastrointestinal symptoms were observed between patients with and without SBI.

Conclusion

The present study demonstrated that the SBI rate was significantly lower in the COVID-19 group than in the non-COVID-19 group. However, the complication rate of SBI, especially UTI, was not negligible, suggesting that at least urinalysis should be performed for target patients. Our study provided more evidence to support the published data pertaining to the risk of SBI in febrile infants with COVID-19.

## Introduction

Febrile infants aged ≤90 days have a higher risk of serious bacterial infection (SBI) than older infants because their immune system is immature [1]. However, it is challenging for healthcare providers to assess the risk of SBI solely on the basis of the infant's appearance. Therefore, "low-risk criteria" have been proposed to determine the level of risk of SBI on the basis of blood and urine test findings. Since the Rochester criteria were published in 1985 [2], various similar criteria have been issued, and in 2000, Baraff et al. [1] published criteria for the management of febrile infants aged 0-90 days. Many Japanese children's hospitals, including our center, use the latter criteria to manage febrile infants aged ≤90 days. Clinically, the infant should be previously healthy, born at term, and have experienced an uncomplicated stay in the nursery. Additionally, the infant must present with a nontoxic appearance and show no signs of focal bacterial infection on physical examination, with the exception of otitis media. In terms of laboratory criteria, the white blood cell (WBC) count should range between 5000 and 15,000/mm³, with fewer than 1500 bands/mm³, or a band-to-neutrophil ratio of less than 0.2. A negative gram stain of unspun urine is preferred, or alternatively, the absence of both urine leukocyte esterase and nitrite, or fewer than 5 WBCs per high-power field (hpf). When diarrhea is present, stool should contain fewer than 5 WBCs per hpf. Regarding cerebrospinal fluid (CSF), the WBC count should be less than 8/mm³, with a negative Gram stain. If the low-risk criteria are not met, inpatient management is actively considered. The same criteria were applied during the years 2020-2022 of the COVID-19 pandemic.

Several previous studies have reported on COVID-19 and the associated risk of SBI in febrile infants aged ≤90 days. Payson et al. [3] reported that infants aged ≤90 days who were positive for SARS-CoV-2 had a significantly lower risk of SBI than their febrile agemates without the infection. On the other hand, in 2021, the American Academy of Pediatrics issued guidelines based on follow-up studies for the management of febrile infants aged ≤60 days [4], which do not mention COVID-19. These guidelines did not provide specific recommendations for infants aged 60 to 90 days. In Japan, there are no reports of an association between COVID-19 and SBI in febrile infants aged ≤90 days, and consequently, no management guidelines for these patients. COVID-19 was first confirmed in Japanese children after February 2020, and the number of cases among children younger than 10 years increased sharply after January 2022 [5].

The present study describes the risk of SBI and clinical characteristics of COVID-19 in febrile infants aged ≤90 days who were seen at the pediatric emergency department of the children's hospital in Japan. Given the lack of specific guidelines on SBI risk in COVID-19-positive febrile infants, this study seeks to provide data to inform clinical decision-making in this population.

## Materials and methods

This retrospective cohort study included febrile infants aged ≤90 days who visited Tokyo Metropolitan Children's Medical Center's emergency department (ED), which houses 561 beds and has an annual intake of 36,000 patients, and was performed between April 2021 and November 2022, when COVID-19 was prevalent among Japanese children. Although variant testing was not conducted at our institution, the predominant SARS-CoV-2 variants circulating in Japan during the study period have been reported as follows. The Alpha variant (B.1.1.7 lineage) became dominant around April 2021 during the fourth wave of infections. Subsequently, the Delta variant (B.1.617.2 lineage, characterized by the L452R mutation) was predominant during the fifth wave from June to December 2021 and was estimated to be approximately 1.5 times more transmissible than the Alpha variant. The Omicron variant (B.1.1.529 lineage) became the predominant strain from January 2022. Throughout 2022, several Omicron sublineages emerged and sequentially replaced one another. Among them, the BA.2 lineage was prevalent in the first half of 2022, followed by a surge of the BA.5 lineage around July 2022, which subsequently replaced BA.2. [6] The study center uses the guidelines proposed by Baraff et al. [1] for the management of febrile infants aged ≤90 days by performing a physical examination followed by a blood, urine, and cerebrospinal fluid (CSF) culture. However, CSF tests and cultures are not mandatory for infants aged >28 days who are in good general condition. The study center places particular emphasis on (1) WBC < 5,000 /μL or > 15,000 /μL, (2) positive urine leukocyte or nitrite reaction, and (3) leukocytes > 5/hpf on urine sedimentation test. Febrile infants meeting any of these criteria have a higher risk of SBI and are admitted. The present study was approved by the institutional review board of Tokyo Metropolitan Children's Medical Center (2022b-70). As this was a retrospective study, the requirement for informed consent was waived by the institutional ethics committee. However, an opt-out process was implemented in accordance with our institution's ethical guidelines, allowing participants the opportunity to decline inclusion in the study.

Patients were extracted from the electronic medical records of all infants aged ≤90 days presenting to the ED during the designated study period. Infants in the target age range presenting to the emergency department with an axillary temperature ≥37.5℃ received a blood and urine test. Diseases requiring surgery and cases deemed inappropriate by the investigator were excluded. Cases excluded by the research physician included those in which the fever at the time of consultation was attributed to causes other than infectious or surgical diseases, and a specific alternative diagnosis was suspected. Specifically, cases such as asphyxiation due to vomiting and milk allergy were excluded. Among the included subjects, the COVID-19 group was defined as those with positive findings for SARS-CoV-2 on polymerase chain reaction (PCR) or those with close contact with a family member who was positive on PCR for SARS-CoV-2. PCR testing was performed using the FilmArray® Respiratory Panel (bioMérieux, Marcy-l'Étoile, France) with nasopharyngeal specimens. Between April 2021 and November 2022, the Tokyo Metropolitan Government implemented two states of emergency and one quasi-emergency measure (man'en bōshi tō jūten sochi) to restrict outings in an effort to prevent the spread of COVID-19. During the state of emergency, people were asked to refrain from going outside, which significantly reduced the frequency of outings and contact with others. As a result, during that time, contact with COVID-19 patients among children was mainly limited to family members living together. Additionally, according to the Infectious Disease Control Law, a quarantine period was mandated for COVID-19, and SARS-CoV-2 antigen or PCR tests were actively conducted for adults with fever. In particular, our study focused on infants under 90 days of age. At this developmental stage, infants do not actively engage with individuals outside their immediate environment, and their contact is typically limited to cohabiting family members. Furthermore, during the pandemic, various measures such as restrictions on going out, visitor limitations, and heightened infection prevention efforts within households were widely implemented. These social conditions support the assumption that infants had limited opportunities for contact beyond their family members living in the same household. Therefore, infants for whom PCR testing was not performed were considered to be at low risk for COVID-19 infection if they did not have a SARS-CoV-2-positive family member living with them. Consequently, participants who did not fall into the COVID-19 group were classified as the non-COVID-19 group.

Vital signs on presentation, symptoms, physical findings, blood test results, urinalysis results, and diagnosis were extracted from the medical records. If a patient was hospitalized, data on oxygen and ventilator use, tracheal intubation, pediatric intensive care unit (PICU) admission, and death were also extracted.

The primary outcome measure was the rate of each SBI type in the two groups. SBI was defined as urinary tract infection (UTI), bacterial pneumonia, bacteremia, or bacterial meningitis [7-9]. UTI, bacteremia, and bacterial meningitis were diagnosed on the basis of blood, urine, and spinal fluid tests and culture findings. Bacterial pneumonia was defined as a diagnosis made by clinicians based on radiographic findings, with antibiotics administered according to pneumonia treatment guidelines.

The secondary outcome measures were the proportion of symptoms, physical findings, treatment, and outcomes of inpatients in the COVID-19 group. Clinical symptoms were compared between the SBI and non-SBI groups in the COVID-19 group. Vital signs, blood test, and urinalysis results, for which data were relatively well available, were compared between the COVID-19 and non-COVID-19 groups. However, due to a large amount of missing data on clinical symptoms and physical examination findings in the non-COVID-19 group, these variables were not compared between the groups. Instead, the characteristics of symptoms and physical findings were presented as proportions for the COVID-19 group only.

Continuous variables (e.g., age) were expressed as the median and interquartile range, and categorical variables (e.g., risk of SBI) were expressed as a number and percentage. Continuous variables were analyzed using the Mann-Whitney U test, and categorical variables were analyzed using Fisher's exact probability test. Two-sided p<0.05 was used to indicate statistical significance. In addition, we calculated 95% confidence intervals (95% CI) to indicate the uncertainty of the estimated effect sizes. EZR version 1.61 was used for all statistical analyses [10].

## Results

**Figure 1:**
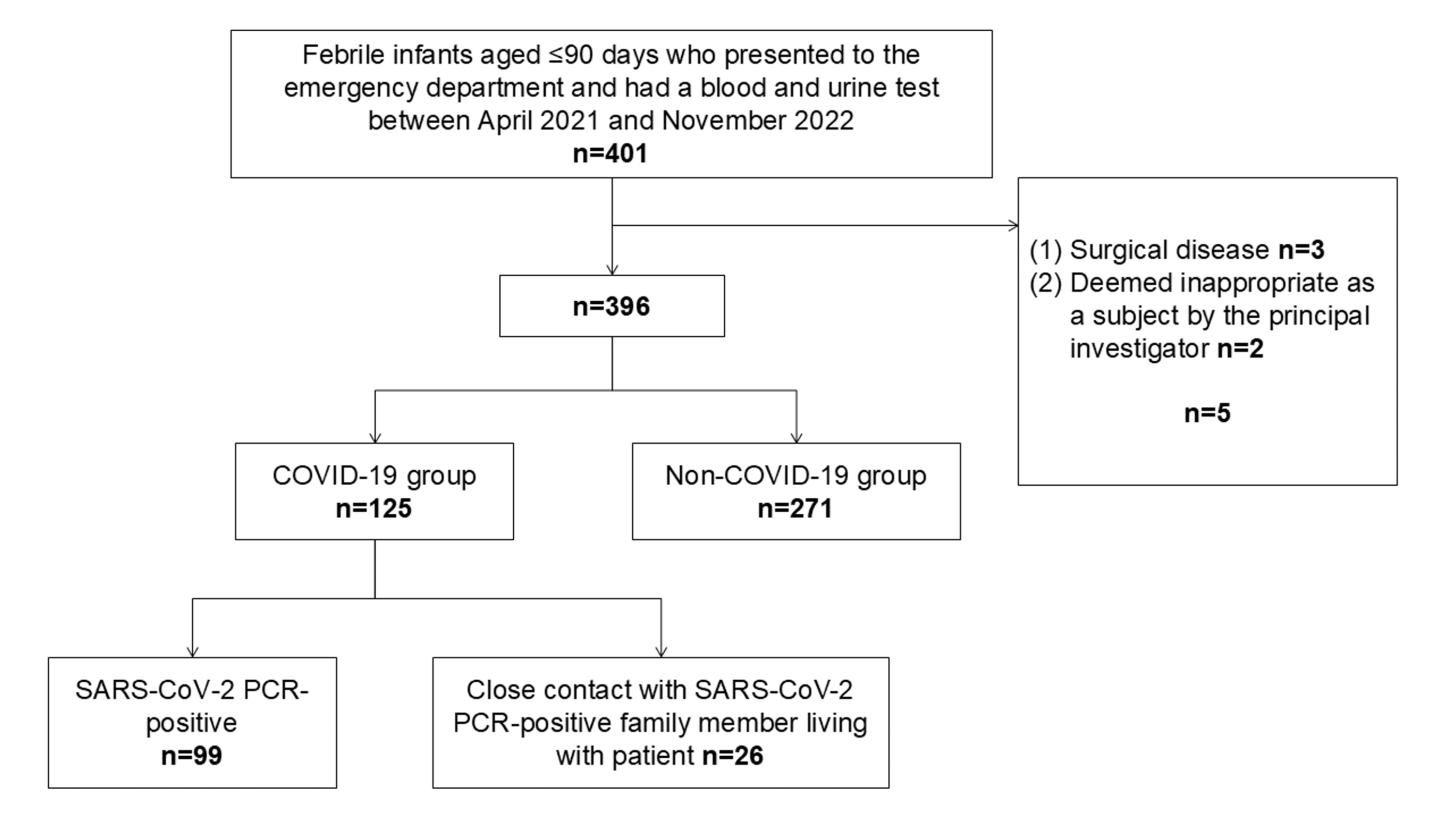
Study flow chart PCR - polymerase chain reaction - SARS-CoV-2 - severe acute respiratory syndrome coronavirus-2

**Figure 2:**
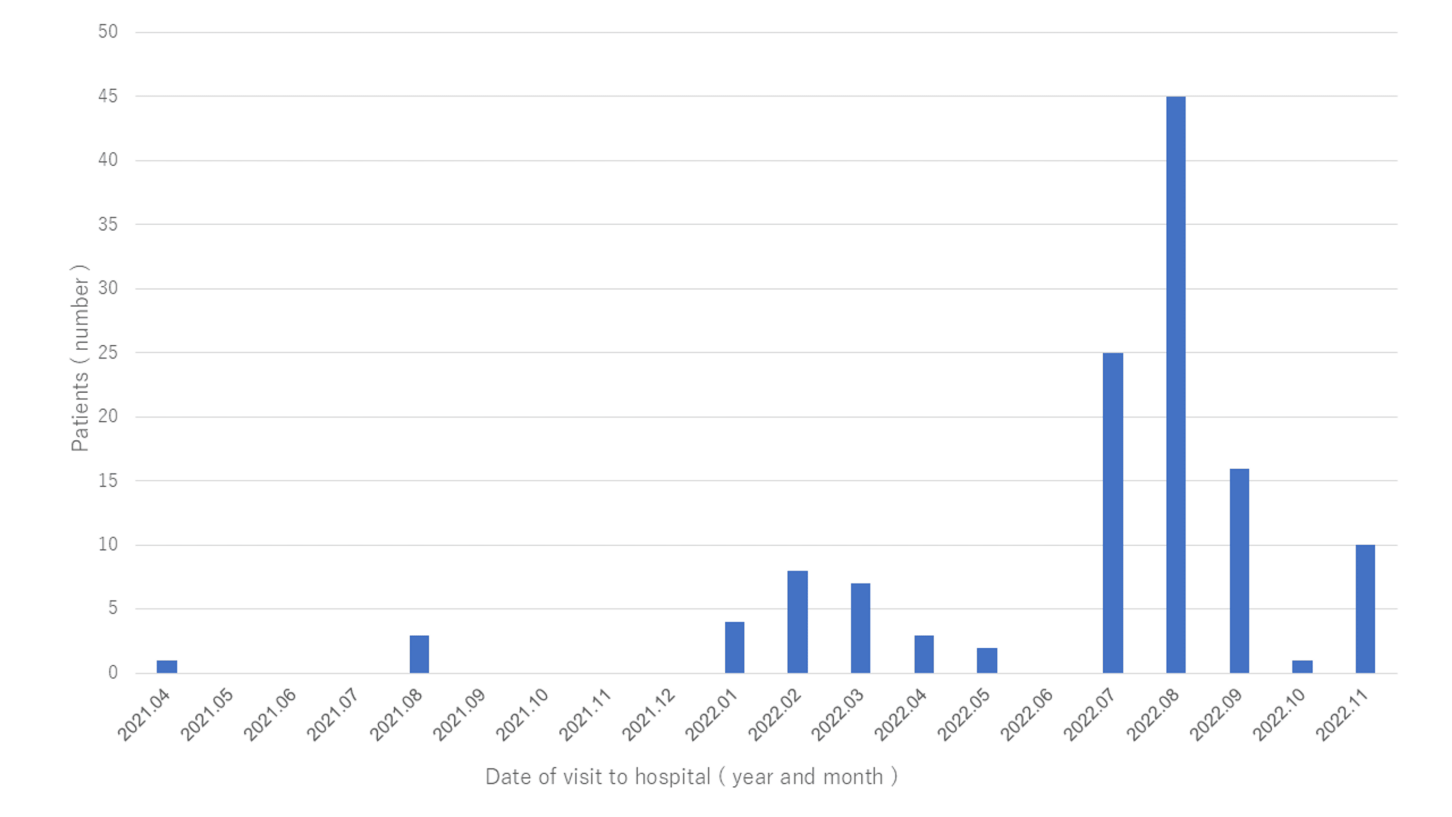
Years and months when patients in the COVID-19 group visited the emergency department at the study center

In total, 396 patients were included; of these, 125 patients were in the COVID-19 group (Figure 1). Figure 2 presents a bar graph summarizing the monthly number of febrile infants under 90 days of age who visited our emergency department during the study period. SBI was diagnosed in seven patients (5.6%) of the group (95% confidence interval (CI): 2.5-11.3%) vs. 45 patients (16.6%) in the non-COVID-19 group (95% CI: 12.6%-21.5%; p<0.05). In the COVID-19 group, all cases of SBI were UTI (Table 1).

**Table 1:** Risk of SBI in the COVID-19 and non-COVID-19 groups Note: All categorical variables are expressed as numbers and percentages. Categorical variables were analyzed using Fisher's exact probability test SBI - serious bacterial infection; UTI - urinary tract infection

	COVID-19 (n= 125）	Non-COVID-19 (n= 271）	p-value
SBI	7 (5.6%)	45 (16.6%)	<0.05
UTI	7 (5.6%)	37 (13.7%)	<0.05
Bacterial pneumonia	0 (0%)	2 (0.7%)	1
Bacteremia	0 (0%)	6 (2.2%)	0.18
Bacterial meningitis	0 (0%)	4 (1.5%)	0.31

In this study, with sample sizes of 125 in the intervention group and 271 in the control group, we observed success rates of 5.6% and 16.6%, respectively. Post hoc power analysis based on these parameters showed a statistical power of approximately 91.7%, indicating that the sample size was sufficient to detect the observed effect.

In the COVID-19 group, 120 patients (96%) had close contact with COVID-19. Table 2 shows their symptoms; in decreasing order of frequency, these were decreased suckling, cough, listlessness, and nasal discharge. Forty-three patients had no symptoms other than fever. Physical findings confirmed by the treating physician included peripheral coldness and livedo reticularis of the extremities. Seventy patients (56%) had no significant examination findings. In the COVID-19 group, there were no significant differences in respiratory symptoms (e.g., nasal discharge, cough) and gastrointestinal symptoms (e.g., vomiting, diarrhea) between the SBI and non-SBI groups (Table 3). Eighty-five patients (68%) were hospitalized. Of the latter, two patients (2%) were admitted to the PICU. There were no deaths (Table 4).

**Table 2:** Clinical characteristics of the COVID-19 group Note: All categorical variables are expressed as a number and percentage. "Sick contact" is defined as close contact with a COVID-19-positive individual. CRT - capillary refill time

COVID-19 (n= 125 patients)	
Sick contact	
Positive	120 (96%)
Negative	5 (4%)
Symptoms	
No symptoms	43 (34%)
Decreased suckling	36 (29%)
Cough	29 (23%)
Listlessness	18 (14%)
Nasal discharge	15 (12%)
Grumpiness	10 (8%)
Vomiting	10 (8%)
Diarrhea	6 (5%)
Pale face	1 (1%)
Convulsions	1 (1%)
Altered consciousness status	1 (1%)
Physical findings	
No findings	70 (56%)
Peripheral coldness	31 (25%)
Livedo reticularis of the extremities	27 (22%)
Skin rash	9 (7%)
Livedo reticularis of the trunk	7 (6%)
Inter/subcostal retraction	4 (3%)
Pharyngeal abnormalities	4 (3%)
Abdominal distention	3 (2%)
Flared nostrils	1 (1%)
Groaning	1 (1%)
Apnea	1 (1%)
Yellowing of the conjunctiva	1 (1%)
Stridor	0 (0%)
Lung murmur	0 (0%)
Decreased consciousness	0 (0%)
Bulging anterior fontanelle	0 (0%)

**Table 3:** Clinical symptoms of the SBI and non-SBI groups in the COVID-19 group Note: All categorical variables are expressed as numbers and percentages. Categorical variables were analyzed using Fisher's exact probability test SBI - serious bacterial infection

	SBI (n= 7)	Non-SBI (n= 118)	p-value
Decreased suckling	1 (14%)	35 (30%)	0.67
Listlessness/ grumpiness	2 (29%)	25 (21%)	0.64
Cough/ nasal discharge	1 (14%)	37 (31%)	0.67
Inter/ subcostal retraction	0 (0%)	4 (3.4%)	1.0
Vomiting/ diarrhea	0 (0%)	15 (13%)	0.60

**Table 4:** Hospitalization in the COVID-19 group Note: All categorical variables are expressed as a number and a percentage. HFNC - high-flow nasal cannula; PICU - pediatric intensive care unit †Abnormal urinalysis was defined as any of the following conditions: (1) positive urine leukocytes, (2) positive urine nitrites, and (3) leukocyte count on urine sedimentation test > 5/hpf.

Hospitalization for COVID-19 (n= 85 patients)	
Reason for hospitalization	
Age ≤28 days	18 (21%)
WBC < 5,000	53 (62%)
WBC ≥ 15,000	5 (6%)
Abnormal urinalysis findings †	7 (8%)
Others	10 (12%)
Treatments & outcomes	
Oxygen only	1 (1%)
HFNC	1 (1%)
Intubation	1 (1%)
PICU	2 (2%)
Death	0 (0%)

Comparison of the blood test results of the two groups (Table 5) revealed that the median WBC was 5,240/µl and 10,770/µl in the COVID-19 and non-COVID-19 group, respectively (p<0.05) while the median CRP value was 0.05 mg/dl and 0.29 mg/dl (p<0.05) for the respective group. Both comparisons indicated a statistically significant difference between the groups. The percentage of patients with WBC < 5,000/μl was 57 patients (46%) and 9 patients (3%) for the respective groups (p<0.05), also indicating a statistically significant difference.

**Table 5:** Vital signs and examination findings of the COVID-19 and non-COVID-19 groups Note: All continuous variables are expressed as the median and IQR. All categorical variables are expressed as a number and a percentage. Continuous variables were analyzed using the Mann-Whitney U test, and categorical variables were analyzed using Fisher's exact probability test. BS - blood sugar; BT - body temperature; CRP - C-reactive protein; CRT - capillary refill time; HCO3- - hydrogen carbonate; hpf - high-power field; HR - heart rate; pCO2 - partial pressure of carbon dioxide; RR - respiratory rate; WBC - white blood cell

	COVID-19 (n=125)	Non-COVID-19 (n=271)	W value	p-value
Patient data				
Age (days)	54 (5-90)	50 (5-90)	18603	0.17
Male sex	64 (51%)	158 (58%)	-	0.19
Vital signs				
HR (beats/min)	171 (120-214)	163 (96-229)	19796	<0.05
RR (min)	45 (26-62)	45 (23-72)	15738	0.55
BT (℃)	38.2 (36.3-39.7)	37.9 (36.5-40.2)	20099	<0.05
SpO2 (%)	98 (90-100)	98 (87-100)	17387	0.49
CRT ≥2sec (n/overall)	26/97 (27%)	63/243 (26%)	-	0.89
Blood test findings				
WBC (/μl)	5,240 (2,660-19,700)	10,770 (3,090-29,050)	16152	<0.05
WBC <5000 /μl	57 (46%)	9 (3%)	-	<0.05
CRP (mg/dl)	0.05 (<0.01-8.76)	0.29 (<0.01-17.57)	11114	<0.05
pH	7.37 (7.22-7.52)	7.37 (7.20-7.51)	16726	0.46
pCO2 (Torr)	40.4 (24.6-54.0)	41.0 (6.2-71.4)	15130	0.41
HCO3- (Torr)	22.3 (17.9-26.1)	22.6 (6.2-30.7)	15100	0.39
Lactate (mmol/L)	2.8 (0.28-7.9)	2.6 (0.56-7.4)	17641	0.1
BS (mg/dl)	97 (59-134)	97 (58-171)	16424	0.66
Urine tests				
Positive leukocytes (n/overall)	5/125 (4%)	24/269 (9%)	-	0.1
Positive nitrites (n/overall)	2/125 (2%)	15/267 (6%)	-	0.11
WBC ≥5/hpf (n/overall)	3/125 (2%)	30/265 (11%)	-	<0.05

## Discussion

To our knowledge, this is one of the largest single-center studies in Japan analyzing SBI risk in febrile infants with COVID-19. The SBI rate in the COVID-19 group was seven patients (5.6%), which was significantly less than the 45 patients (16.6%) in the non-COVID-19 group. All COVID-19 patients with SBI had UTI, and none had bacterial meningitis, bacteremia, or bacterial pneumonia.

In their retrospective study of febrile children aged ≤90 days, Payson et al. [3] found the risk of SBI to be four patients (8%) in a COVID-19 group comprising 53 patients and 18 patients (34%) in a control group of 53 patients (relative risk: 0.22). The most common SBI in the former was also UTI (6%; 3/53 cases), and there were no cases of bacteremia or bacterial meningitis. Although the overall SBI rate was slightly higher in their study [3] than in ours, both found a statistically significant difference in the risk of SBI between the COVID-19-positive and negative groups, with the most common SBI in the COVID-19 group being UTI. A previous, retrospective study by Guernsey et al. (enrolling 62 and 171 infants aged ≤60 days who were positive and negative for SARS-CoV-2, respectively) [7] and a multicentric, cross-sectional study by Aronson et al. (enrolling 3753 and 10,649 infants aged ≤60 days who were positive and negative for SARS-CoV-2, respectively) [8] reported that the risk of SBI in the COVID-19 group was 1.0-1.7%, which differed significantly from the risk in the non-COVID-19 group. The most common SBI in the COVID-19 group was UTI.

Several previous studies have reported complications of infectious respiratory viral infections other than SARS-CoV-2, such as the influenza virus and respiratory syncytial virus (RSV), and SBI in febrile infants aged ≤90 days [11-12], and although respiratory virus-positive patients have lower SBI rates than negative patients. These studies consistently demonstrate that respiratory virus-positive infants have significantly lower rates of SBI compared to virus-negative infants. However, urinary tract infections (UTIs), in particular, remain a notable concern even in virus-positive cases. Therefore, SBI should not be excluded on the basis of respiratory viral multiplex PCR results alone because the risk of SBI is non-negligible [9, 11-13]. Where the influenza virus was concerned, the SBI rate was 2.5%, and the UTI rate was 2.4% [11]. In patients with an RSV infection, the SBI rate was 7.0%, and the UTI rate was 5.4% [12]. The present study found that COVID-19 was associated with a lower risk of SBI than other viral infections, as reported in previous reports. However, two previous reports described this risk as non-negligible [11-12]. In this study, a clinically significant proportion of febrile infants with COVID-19 were diagnosed with UTI. We found no significant differences in respiratory symptoms between those with and without SBI, indicating that the presence of respiratory symptoms does not help distinguish infants with UTI from those without. Therefore, clinicians should not rule out UTI based solely on positive respiratory viral multiplex PCR results or a known exposure to respiratory viruses. Urine testing should be performed, at a minimum, in all febrile infants aged ≤90 days with COVID-19, regardless of respiratory findings. 

COVID-19 is less severe in children, where it is often asymptomatic or manifests non-specific symptoms, such as fever and cough [14-15]. In the present study (Table 3), the most frequently observed symptoms were non-specific, such as decreased suckling and cough. The most common physical findings were peripheral coldness and livedo reticularis of the extremities, which are also non-specific in young infants. A systematic review of COVID-19 infants aged ≤90 days reported that most had mild to moderate disease and were cured with symptomatic treatment alone [16]. In the present study, 85 (68%) of the patients in the COVID-19 group were hospitalized. Of these, only two patients (2%) were admitted to the PICU. Ozdemir et al. [17] reported that COVID-19 in neonates was associated with a lower rate of oxygen support than RSV. Children, including young infants, have a lower risk than adults for severe COVID-19 infection [15] and should be treated as for a common viral infection in the context of appropriate infection control measures.

Significantly more patients in the COVID-19 group had a WBC count of <5000/μl (p<0.05). One of the admission criteria of the study center includes a WBC count of <5000; therefore, the hospitalization rate in the COVID-19 group was as high as 68%. Two meta-analyses [18-19] reported that most children with COVID-19 had a normal WBC count, and that the most common abnormality was leukopenia. In the 2021 American Academy of Pediatrics (AAP) guidelines [3], the lower limit of the WBC count was not adopted as a low-risk criterion due to a lack of evidence. The admission criteria at the study center include a lower limit for leukocytes, following the criteria of Baraff et al. [1]. In febrile infants with COVID-19, hospitalization may not be necessary if the other findings are normal and UTI is not suspected, even if the WBC count is low.

The present study has several limitations. First, it was monocentric. The study center is an advanced critical care center specializing in pediatric medicine; therefore, there was a selection bias in that pediatric patients with COVID-19 requiring hospitalization were more likely to be admitted. Second, respiratory viral multiplex PCR was not used in all the cases. In principle, PCR testing was not performed for outpatients with a mild illness who did not require hospitalization. In infants who were returned home, COVID-19 was diagnosed on the basis of symptoms, such as fever, and contact with infected individuals. Third, if the PCR result was positive, it was not possible to distinguish whether the patient had active COVID-19 or whether the PCR merely detected residual viral material from a previous infection. Since we did not perform quantitative viral load testing, we could not precisely evaluate the timing of infection. However, given the patients' age (all younger than 90 days), the likelihood of prior infection was considered low. Fourth, we did not systematically differentiate COVID-19 from other viral infections. Among the hospitalized cases in which multiplex PCR testing was performed, a few cases of viral co-infection were identified - two with RSV and three with rhinovirus/enterovirus. No cases of hMPV or influenza were detected. However, considering the clinical presentation, the presence of household contacts with confirmed COVID-19, and the epidemiological context, COVID-19 was considered the primary infection. Fifth, among infants with COVID-19 who were diagnosed with SBI, only UTI was identified. This limits our ability to assess the risk of other types of SBIs, such as bacteremia or meningitis, in this population. Finally, the small number of SBI cases in the COVID-19 group may limit the ability to properly assess statistical significance. As a result, the absence of significant findings does not necessarily indicate the absence of a difference. This limitation should be considered when interpreting the results.

## Conclusions

To the best of our knowledge, the present retrospective cohort study was the first in Japan to include as many as 125 febrile infants aged ≤90 days with COVID-19. The present study demonstrated that the SBI rate was significantly lower in the COVID-19 group than in the non-COVID-19 group of febrile infants aged ≤90 days. However, SBI cases still occurred in the COVID-19 group, indicating that the presence of COVID-19 does not entirely rule out the possibility of SBI. Since there was no significant difference in symptoms between the SBI and non-SBI groups within the COVID-19 group, and the complication rate of SBI, especially UTI, was not negligible at 5.6% (7/125 patients). Urinalysis should be performed at least in febrile infants aged ≤90 days, even if COVID-19 is suspected as the source of the fever.
